# Distribution of pharmaceuticals in marine surface sediment and macroalgae (ulvophyceae) around Mombasa peri-urban creeks and Gazi Bay, Kenya

**DOI:** 10.1007/s11356-024-35881-4

**Published:** 2025-01-24

**Authors:** Veronica Wayayi Ogolla Wanjeri, Eric Okuku, Jane Catherine Ngila, Josephine Ouma, Patrick Gathura Ndungu

**Affiliations:** 1 Department of Chemical Sciences, University of Johannesburg, Johannesburg, South Africa; 2 Kenya Marine and Fisheries Research Institute, P.O. Box 81651, Mombasa, Kenya; 3 Department of Chemistry, University of Pretoria, Hatfield, Pretoria, South Africa; 4 Jomo Kenyatta University of Agriculture and Technology, P.O. Box 62000, Nairobi, Kenya

**Keywords:** Pharmaceuticals, Coastal areas, Surface sediment, Peri-urban creeks, Macroalgae, Ulvophyceae

## Abstract

Pollution in marine creeks has been increasing due to anthropogenic activities and has been a global concern. Limited research has been conducted on pharmaceuticals in marine sediment and macroalgae in African countries. In the present study, the levels of pharmaceuticals were assessed in surface sediment and different species of macroalgae (*ulvophyceae*; *Cladophora sudanensis*, *Chaetomorpha crassa*, *Chaetomorpha indica*, *Enteromorpha kylinii*, *Ulva reticulate*, *Ulva lactuca* and *Cladophora sibugae*) in Mombasa peri-urban creeks (Tudor, Makupa and Mtwapa creek) and Gazi bay during dry and wet seasons. The concentration of pharmaceuticals in the surface sediment during dry and wet seasons ranged between 0.04–686.8 ng/g and 0.01–2580.6 ng/g, respectively. The highest concentration of pharmaceuticals was observed in Tudor creek in dry and wet seasons, with a sum concentration of ∑1013 ng/g and ∑3111 ng/g, respectively. Gazi Bay was used as a reference environment for this study, and pharmaceuticals were detected in dry and wet seasons with a concentration of 0.10–686.8 ng/g and 0.18–93.5 ng/g, respectively. There was no seasonal variation in the pharmaceutical concentration during the dry and wet seasons. For macroalgae of *ulvophyceae* species, pharmaceutical concentration ranged below the detection limit (DL)–11059 ng/g. Compared to surface sediment, macroalgae showed a high concentration of pharmaceuticals in Tudor, Makupa and Mtwapa creeks. Tetracycline was higher in sediment and macroalgae in all the creeks than other pharmaceutical compounds. A high concentration of pharmaceuticals confirms sewage effluent into the marine environment of Mombasa peri-urban creek. Pharmaceuticals (acetaminophen, sulfamethoxazole, trimethoprim, carbamazepine and nevirapine) in sediment posed a medium (− 1 < Log10 RQ < 0) to higher risk (Log10 RQ > 0) to algae, invertebrates and fish in the dry and wet seasons. There is a need to sensitise the residents of coastal cities on the impact of sewage effluent into the marine environment and enact strict measures to limit the discharge of sewage effluents containing these contaminants into the marine environment. Nevertheless, it is recommended to conduct further research on the distribution of pharmaceuticals in the marine environment and the long-term combined impacts of these substances of these compounds on marine biota.

**Supplementary Information:**

The online version contains supplementary material available at 10.1007/s11356-024-35881-4.

## Introduction

Coastal areas, especially creeks and lagoons, receive large amounts of contaminants due to their location between land and sea, making them the most vulnerable marine environments to marine pollution (Carafa et al. 2007; Moreno-González et al. 2016). The occurrence of pharmaceuticals in aquatic environments has received considerable attention in recent years due to their high consumption (Chaves et al. 2022; Kötke et al. 2019), frequent detection in the environment, reported (eco) toxicological effects (Minguez et al. 2016) and extensive and long-term use in human and veterinary medicine (Zhang et al. 2018). This is exacerbated by the ever-growing global population and the increased demand for pharmaceuticals that can treat new diseases or provide improved treatment options for known ailments (Lolić et al. 2015). Pharmaceuticals enter aquatic environments through the discharge of treated or untreated wastewater originating from various sources. Such sources can include point and non-point discharges from pharmaceutical production and processing facilities, agricultural and animal husbandry, aquaculture, hospitals or informal settlements (Biel-Maeso et al. 2017; Roveri et al. 2020; Ulvi et al. 2022; Xie et al. 2019). They are mobile in the aqueous phase due to their hydrophilic nature aiding their transportation (Brumovský et al. 2017). However, these compounds can also interact with particulate matter in suspension and transfer into the sediment (Beretta et al. 2014; Fabbri and Franzellitti 2016; Moreno-González et al. 2016). Sediment thus acts as a temporary sink and reservoir of pharmaceuticals and as a secondary source that releases pharmaceuticals when environmental conditions such as salinity and pH or during tidal changes or storm events (Fabbri and Franzellitti 2016; Fernandes et al. 2020; Gaw et al. 2014; Siedlewicz et al. 2018).

The continuous discharge of these compounds into the marine ecosystem can result in continuous exposure and accumulation of pharmaceuticals in benthic aquatic organisms (Ali et al. 2018). For many years, macroalgae have served as biomonitors for monitoring coastal pollution. Unlike sessile organisms, they possess the ability to readily respond and adapt to sudden environmental changes. Additionally, their widespread presence in nearly all coastal areas makes them a representative sample for various conditions (Ali et al. 2018; Rodríguez-Romero et al. 2021; Świacka et al. 2022). As primary producers, algae play an important role in contamination along the food chain due to their ability to bioaccumulate contaminants and pass them to higher trophic levels (Noaman and Zaky 2017). Macroalgae possess cell wall structures that contain various polysaccharides such as cellulose and alginate. These polysaccharides contain carboxylic acid, hydroxyls and amine-based chemical functional groups, which contribute to the macroalgae’s strong affinity and selectivity towards organic compounds (Nazal et al. 2021). The capacity of macroalgae to bio-accumulate pharmaceuticals has not been widely investigated in the marine environment since the prevailing view is that hydrophilic/lipophilic interactions rule the biological uptake of pharmaceuticals. Thus, organic compounds are more likely to attach to sediments or chemically bond to fatty tissues present in fish compared to algal cells (Moreno-González et al. 2016; Rodríguez-Varela et al. 2021). However, the case of bioaccumulation of pharmaceuticals in marine macroalgae has been reported in coastal areas in Europe (Álvarez-Muñoz et al. 2015) False Bay, South Africa (Ojemaye and Petrik 2021) and coastal waters of the Saudi Red Sea (Ali et al. 2018).

Several studies have been carried out to evaluate the occurrences and distribution of pharmaceuticals in marine sediments in Santos Bay, Brazil (Beretta et al. 2014); Mediterranean coastal lagoon, SE Spain (Moreno-González et al. 2015); Bohai Bay, China (Cheng et al. 2016); Southern Baltic Sea (Siedlewicz et al. 2016); Tokyo Bay, Japan (Tsui et al. 2015); and Augusta Bay, Southern Italy (Feo et al. 2020). The concentration of pharmaceuticals in marine sediment has been reported to range from ng/g to µg/g, depending on the compound, local waste and wastewater discharge practices, and environmental conditions such as water exchange and sediment composition. However, despite the rapidly increasing consumption of pharmaceuticals, only a few studies have been conducted on the level of pharmaceuticals in marine sediment on the African continent; one such example includes a study done at False Bay in Cape Town, South Africa (Ojemaye and Petrik 2021).

The Kenyan government expenditure on health has increased from 6.1% in 2012/2013 to 6.7% in 2015/2016 (Republic of Kenya 2017), with a number of studies reporting the presence of pharmaceuticals distributed in freshwater (Bagnis et al. 2020; K'oreje et al. 2012; Kairigo et al. 2020; Ngumba et al. 2016). However, there is limited information on the distribution and level of pharmaceuticals in sediment and macroalgae in marine aquatic environments. This study therefore aimed at determining the levels and distribution of pharmaceuticals in sediment and macroalgae in pollution impacted Mombasa peri-urban creeks and relatively pristine reference site (Gazi Bay).

## Methodology

### Study area

**Fig. 1:**
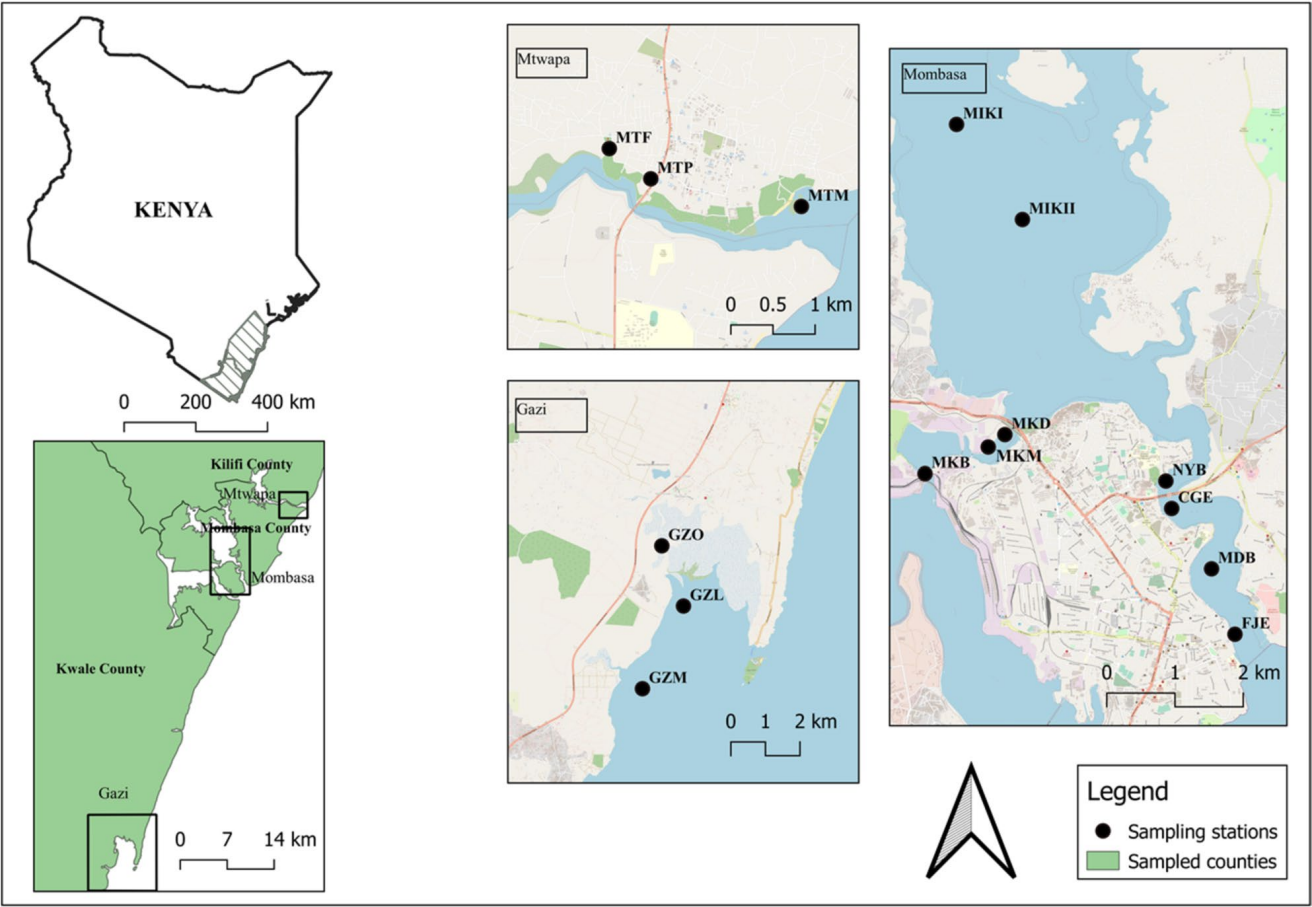
Map showing sampling stations in Tudor creek (FJE, MDB, CGE, NYB, MIKII and MIKI), Mtwapa creek (MTF, MTP and MTM), Makupa creek (MKM, MKB and MKD) and Gazi Bay (GZO, GZL, and GZM)

Sampling was conducted in different locations along Mombasa’s peri-urban creeks (Tudor, Makupa and Mtwapa creeks) and Gazi Bay (Fig. 1). The sampling sites were identified, and coordinates recorded using a hand-held Global Positioning System (GPS) (Model: GARMIN GPS-12). In Mombasa peri-urban creek, untreated domestic and industrial wastewater and chemical residues are discharged due to the absence or malfunctioning of proper wastewater management and sanitation facilities (Wanjeri et al. 2023).

Makupa and Tudor creeks are west and east of Mombasa Island. The port of Mombasa, one of the largest ports on the East African coast, is located on Mombasa Island. Besides maritime traffic and activities, other notable anthropogenic contributions to pollutants to the creeks are likely due to surrounding landfills, domestic and industrial effluents, and informal settlements (Kamau et al. 2015). About 25 km north of Mombasa lies Mtwapa creek, which is characterised by mud banks and mangrove forests. The most notable discharge into this tidal creek is sewage from the nearby Shimo la Tewa prison (Okuku et al. 2019). Finally, 50 km to the south of Mombasa is the tropical lagoon of Gazi Bay which was used as a reference sampling site. Gazi Bay is located near a remote village with mangrove forests, large seagrass beds and fringing coral reefs (Nyunja et al. 2009).

### Sample collection and preparation

Sediment and macroalgae samples were collected during the wet season (June 2021) and the dry season (December 2021). Replicate grab surface sediment samples were collected using a Van Veen grabber and then stored in pre-cleaned aluminium foil. Macroalgae samples of different species were individually collected by picking, then wrapped in aluminium foil and labelled. The sediment and macroalgae samples were placed in a cooler box (4 °C), transported to the laboratory and stored at − 20 °C before analysis. All the sediment and macroalgae samples were freeze-dried to a constant weight and homogenised using a mortar and a pestle. The sediment samples were then sieved through < 200 µm to remove gravel-sized materials, leaves and other particles prior to analyses.

### Chemicals and standards

High-performance liquid chromatography (HPLC)-grade methanol and acetonitrile (Merck), hydrochloric acid (HCl), sulfuric acid (H_2_SO_4_), potassium dichromate (K_2_Cr_2_O_7_) and ethylenediaminetetraacetic acid disodium salt dihydrate (Na_2_EDTA) were purchased from Sigma–Aldrich Ltd. Formic acid (99 + % purity) was purchased from Thermo Scientific, South Africa. All standards were of high-purity grade (> 99%). The following pharmaceutical standards were purchased from Sigma-Aldrich Ltd (South Africa): acetaminophen, ibuprofen, caffeine, tetracycline, acetylsalicylic acid, lidocaine and bupivacaine. The standards purchased from Toronto Research Chemical included erythromycin, diclofenac, carbamazepine and trimethoprim. The Universal Corporation Ltd, Kenya, kindly donated sulfamethoxazole and nevirapine (99%), and cetirizine dihydrochloride was purchased from Dr. Ehrenstorfer GmbH.

For internal standards, isotopically labelled carbamazepine d_10_, diclofenac d_4_ and trimethoprim ^13^C_3_ were acquired from Toronto Research Chemicals (Ontario, Canada). Individual stock standards and isotopically labelled internal standards were prepared in methanol (at a concentration of 1000 mg/L) and stored at 4 °C in the dark. Working standard solutions containing all pharmaceuticals were also prepared in methanol/water (50:50, v/v). Separate mixtures of isotopically labelled internal standards were prepared in methanol for internal standard calibration, and further dilutions were made in a methanol/water mixture (50:50, v/v). The solid-phase extraction (SPE) cartridges, oasis hydrophilic-lipophilic balanced (HLB; 6 cm^3^, 500 mg), were purchased from Waters (Milford, USA).

### Sample characterization

A Malvern Mastersizer 3000 particle size analyser was used to examine the sediment grain size. The percentage distribution of sediment particles was categorised into three groups based on the Wentworth grain size classification (Wentworth 1922): clay (< 3.9 µm), silt (3.9–62.5 µm) and sand (> 63 µm). Approximately 10 g of the sediment samples were combusted in the furnace at 450 °C for 6 h to determine the organic matter content in the sediment samples. Total organic carbon content (%TOC) was measured by wet oxidation with K_2_Cr_2_O_7_, after pretreatment of the sediment with H_2_SO_4_ to remove inorganic carbon (FAO 2020).

### Sediment and macroalgae sample treatment and analysis

Sediment samples were analyzed using the Environmental Protection Agency (EPA) method 1694 United States Environmental Protection Agency (USEPA) (2007), with minor adjustments. In brief, about 1 g of sediment samples were weighed into a 50-mL borosilicate glass sample vial and 5 mL phosphate buffer and mixed by a vortex for 5 s. The sample was spiked with 50 µL of 100 ng/L mixed internal standard and pharmaceuticals extracted using methanol: acetonitrile (2:1) in an ultrasonic bath at 25 °C for 15 min. Samples were then centrifuged (4000 rpm) for 5 min, and the supernatant was transferred into a glass beaker. The extraction procedure was repeated twice, after which the collected supernatant solutions were combined. In order to chelate the metal ions in the solution, 0.5 g of Na_2_EDTA and 200 mL of ultrapure water were added to the combined extract. The samples were then passed through Oasis HLB (6 cm^3^, 500 mg) SPE at a 5-mL/min flow rate. To dry the cartridges, a vacuum was then applied for 30 min, and then, the analytes were eluted with 3 mL acetonitrile/methanol (50:50 v/v). The solvent was then evaporated to dryness using Genevac EZ-2, and the samples were then reconstituted to 100 µL with HPLC-grade water and methanol (50:50 v/v), and injected into a liquid chromatography system hyphenated to mass spectrometry detector.

Macroalgae samples were extracted following the method Klosterhaus et al. (2013) and USEPA (2007) described. In brief, approximately ~ 0.5 g (dry weight; dw) of each macroalgae sample was weighed in a 50-mL borosilicate glass sample vial and spiked with 50 μL of 100 ng/L internal standards followed by the addition of 10 mL of methanol and acetonitrile in a ratio of 2:1 was added in a 50-mL borosilicate glass vial. The resulting mixture was vortexed for 2 min and sonicated at 25 °C for 15 min. Samples were then centrifuged (4500 rpm) for 10 min, and the supernatant was transferred to a borosilicate glass vial. This extraction procedure was repeated twice with methanol and acetonitrile, followed by phosphate buffer. Thereafter, the extractions were combined and evaporated using Genevac EZ-2. Immediately after concentration, the extract was diluted with 200 mL of ultrapure water (18.2 MΩ × cm at 25 °C), pH adjusted to 2, followed by adding 0.5 g Na_2_EDTA to chelate the metal ions in the solution. The samples were passed through an SPE cartridge (Oasis HLB, 6 cm^3^, 500 mg) at a 1-mL/min flow rate. Reagent water (10 mL) was added to rinse the Na_2_EDTA after the entire sample had passed through the SPE cartridge. Using a vacuum (30 min), the cartridges were dried, and then the analytes were eluted with 3 mL acetonitrile/methanol (50:50 v/v). The solvent was evaporated to dryness using Genevac EZ-2, and then reconstituted to 100 µL with HPLC-grade water and methanol (50:50 v/v). Samples were then injected into a liquid chromatography system hyphenated with a mass spectrometry detector.

### Liquid chromatography–mass spectrometry analysis

The liquid chromatography system with mass spectrometry detector used in this study was an Acquity Ultra Performance Liquid Chromatography (UPLC®) system, hyphenated with a quadrupole-time-of-flight (QTOF) instrument (Waters® Synapt G2), for compound separation and detection. To maintain mass accuracy, a solution of leucine enkephalin (*m/z* 555.2693), with a concentration of 2 ng/μL, was infused directly into the source through a secondary orthogonal electrospray ionisation (ESI) probe. This internal lock mass control standard compensated for instrumental drift during the runs. The calibration of the UPLC-QTOF was done using sodium formate clusters and Intellistart functionality (mass range 112.936–1132.688 Da). The mass error for the system was within 0.4 mDa, and a resolution of 20,000 at *m/z* 200 (full width at half maximum).

The source conditions for ESI were as follows: 2.6 kV for positive mode ionisation (acetaminophen, trimethoprim, sulfamethoxazole, erythromycin, carbamazepine, nevirapine, caffeine bupivacaine and lidocaine) and 2.0 kV for negative mode ionization (diclofenac, ibuprofen, acetylsalicylic acid and tetracycline). The sampling cone voltage was set to 25 V, and the source temperature was set at 120 °C. The extraction cone voltage was set at 4.0 V, and the nitrogen (cone gas) flow rate was set at 10.0 L/h. The desolvation temperature was set at 350 °C with the nitrogen gas flow rate set at 600.0 L/h. Two simultaneous acquisition functions with low and high collision energy (MSE approach), on the QTOF instrument, were used for quantitative data-independent acquisition. The high-energy mass spectral scan was time-aligned with the low-energy scan to obtain the full mass spectrum. Fragmentation patterns were utilised for qualitative confirmation using high-energy collision-induced dissociation with fragmentation energy set at 2 V and 3 V for the trap and collision energy, respectively. Mass spectral scans were collected every 0.3 s, covering a mass range of m/z 50 to 1200 Da.

On the UPLC, a reverse-phase step gradient elution was used for separation, starting with 95% H_2_O (0.1% formic acid), and ending with 100% methanol (0.1% formic acid). The gradient started with an isocratic flow, followed by a linear increase to 100% methanol, with subsequent column washing, conditioning and equilibration before the start of the next run. The flow rate was set at 0.4 mL/min, and the column temperature was kept at 40 °C. The total run time for each sample was 20 min. Sample injection volumes were 5 µL. The column used on the UPLC was a Kinetex® 1.7 µm EVO C18 100 Å (2.1 mm ID × 100 mm length). Separate chromatographic runs were performed to collect positive and negative ion mass spectra. The pharmaceutical target compounds were identified through qualitative comparison of their retention times and corresponding spectra with those of standard solutions.

### Quality control and assurance

Procedure blanks were established for each batch to evaluate potential contamination during the experiment. Seven standard solution concentrations (5–1000 ng/L) of individual pharmaceuticals were used to calculate the calibration curves. Any samples with concentration values that exceeded the calibration range were diluted (10 ×), then reinjected, and the dilution factor was taken into account when calculating the final concentration. All the calibration curves had *r*^2^ values above 0.990. Instrumental inter-day repeatability was evaluated by spiking sediment and macroalgae (*n* = 6) with all target analyte. The calculated relative standard deviation (RSD) ranged between 1.25%–19.20% and 1.01%–19.49% in sediment and macroalgae, respectively. Accuracy was determined in triplicate by comparing the concentrations found in pre-spiked samples (100 μL of 20 ng/L standard mix) with post-spiked samples, which varied from 75 to 105% and 72 to 95% in sediment and macroalgae, respectively. Methanol blanks were also run between samples to monitor instrumental contamination and carryover. To evaluate the sensitivity of the method, both the limit of detection (LOD) and the limit of quantitation (LOQ) were calculated. The LOD and LOQ represent the minimum detectable amount of the analyte with signal-to-noise ratios of 3 and 10, respectively (Table 1). An estimation of uncertainty bias (*u*_*b*_) was investigated in method validation and calculated using Eq. (1) as described by Boleda et al. (2013).

1 $${u}_{b}=\sqrt{{u}_{{RV}^{2}}+{u}_{{SD}^{2}}+{u}_{{corr}^{2}}}$$

where *u*_*RV*_ is the uncertainty estimate for the reference value used (concentration (ng/L) of the analyte in the fortified sample), *u*_*SD*_ is the uncertainty obtained from the precision of the mean value of replicate measurements and *u*_*Corr*_ is the uncertainty of the corrected analyte content (related to recovery) (Boleda et al. 2013). The uncertainty values in sediment and macroalgae ranged between 0.3 ng/g to 3.9 ng/g and 0.1 ng/g to 3.4 ng/g, respectively.

**Table 1:**
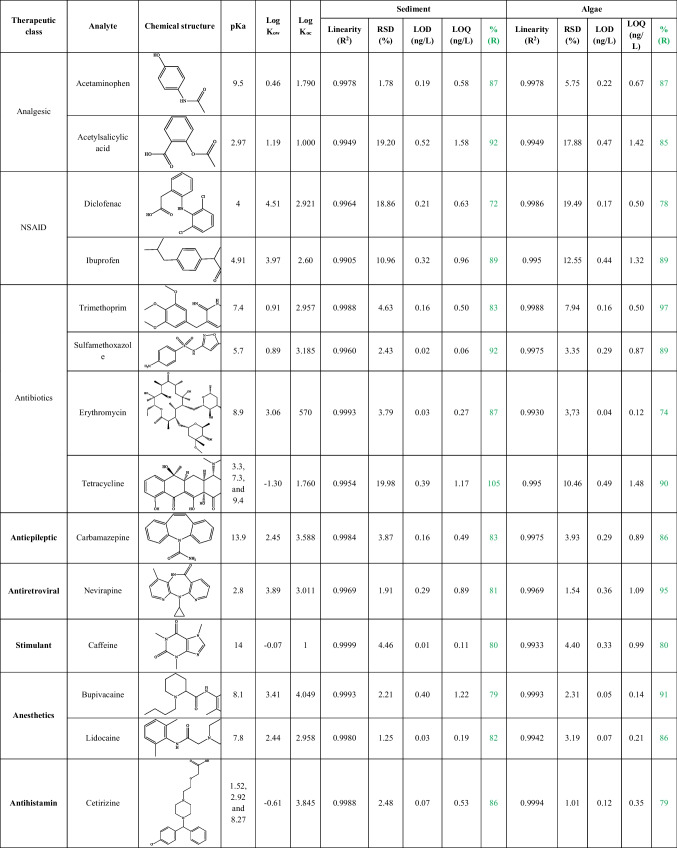
Chemical structure of targeted pharmaceutical, calibration, relative standard deviation (RSD), % Recovery (%R), limit of detection (LOD) and limit of quantification (LOQ) for individual analytes

pK_a_: dissociation constant;. Log K_ow_: octanol–water partition coefficient;

### Calculation of the bioconcentration factor (BCF)

The BCF was used to estimate the uptake and enrichment of substances from the surrounding water by macroalgae using Eq. (2). The estimated concentrations of five pharmaceuticals (acetaminophen, sulfamethoxazole, trimethoprim, carbamazepine and nevirapine) in coastal waters were obtained from data reported by Wanjeri et al. (2023) and were subjected to BCF calculations.

2 $$BCF=\frac{{C}_{b}}{{C}_{w}}\times 1000$$

The term *C*_*b*_ (ng/g) is the concentration of pharmaceuticals in the macroalgae samples, *C*_*w*_ (ng/L) is the concentration of pharmaceuticals in water samples and the unit of BCF is expressed as L/kg (Xie et al. 2019; Zhang et al. 2018). Chemicals are classified as “bioaccumulative” when their bioconcentration factor (BCF) exceeds 5000 L/kg, and as “potentially bioaccumulative” when their BCF ranges from 2000 to 5000 L/kg in biota samples (Zhang et al. 2018).

### Potential environmental risk assessment

The risk quotient (RQ) approach was used to evaluate the potential ecological risks of pharmaceuticals in sediment. The RQ values for pharmaceuticals in sediment were determined using Eqs. (3) to (5) (Kondor et al. 2022; Peng et al. 2020). Among the 14 targeted pharmaceutical compounds, *PNEC*_*marine water*_ values (Tables S1 and S2) of only five pharmaceuticals (acetaminophen, sulfamethoxazole, trimethoprim, carbamazepine and nevirapine) could be estimated using marine water data reported by Wanjeri et al. (2023) and the results were then used in the calculation *PNEC*_*sediment*_ using Eq. (4)

3 $${PNEC}_{marine water}={EC}_{50}/AF$$

4 $${PNEC}_{sediment}=\frac{{PNEC}_{marine water}}{1000}\times \frac{{K}_{oc}}{\%TOC}$$

5 $$RQ=MEC/{PNEC}_{sediment}$$

where *MEC* stands for the measured environmental concentration, *PNEC*_*marine water/sediment*_ represents the non-effect concentration predicted, EC50 is indicative of the half-maximal effective concentration and *PNEC* was estimated considering the EC50 for short-term standard toxicity studies using the most sensitive species among three trophic levels (algae, invertebrates and fish), and applying an assessment factor (AF) value of 1000. Koc denotes the sediment–water partition coefficient. According to the model reported by Peng et al. (2020), the Koc value was calculated based on the octanol–water partition coefficient (Kow) of each target compound (Table 1). Therefore, potential ecological risks of sedimentary pharmaceuticals were classified into no risk (Log10 RQ <  − 2), low risk (− 2 < Log10 RQ <  − 1), medium risk (− 1 < Log10 RQ < 0) and high risk (Log10 RQ > 0) according to the Technical Guidance Document on Risk Assessment (EC 2013). Detailed information about the RQ for each detected pharmaceutical is presented in the supplementary materials (Table S1).

### Statistical analysis

Descriptive statistics were calculated using Microsoft Excel 2016 and used to summarise the results of pharmaceuticals and physicochemical variables in surface sediment and macroalgae samples. The data was plotted using origin 8.5. Correlations analysis between pharmaceuticals and the physiochemical parameters of sediment and individual pharmaceuticals were investigated using R studio.

## Results and discussion

### Occurrence of pharmaceutical residue in sediment

**Fig. 2:**
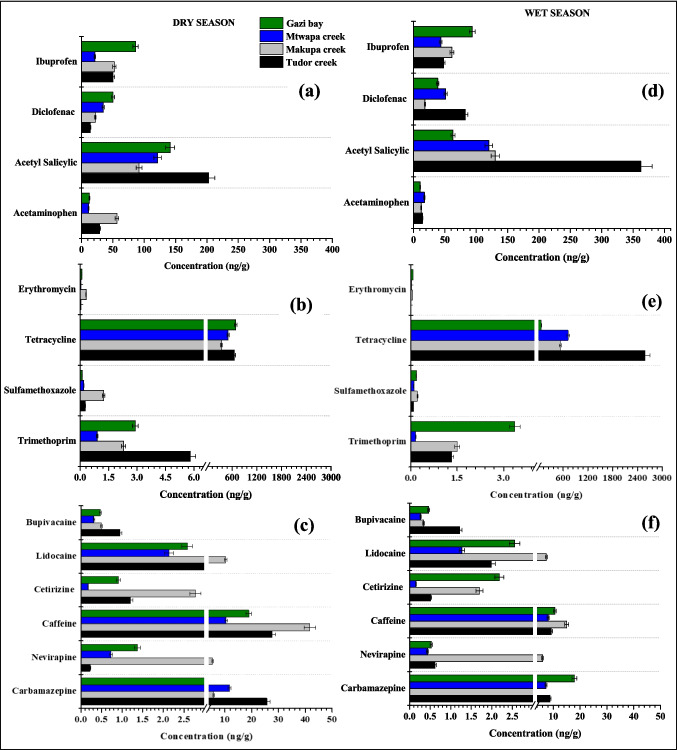
Mean concentration (± SD) of pharmaceuticals in surface sediment (ng/g dry weight) collected from Tudor, Makupa, Mtwapa creek, and Gazi Bay during dry (panels (**a**) – (**c**)) and wet (panels (**d**) – (**f**) seasons. Note there is a non-linear break included on the concentration scale for panels (**b**), (**c**), (**d**), and (**e**) due to the large differences in concentration

The target pharmaceutical concentration in the surface sediment during dry and wet seasons ranged between 0.04–686.8 ng/g and 0.01–2580.6 ng/g, respectively. In the dry season, Tudor, Makupa, Mtwapa creeks and Gazi Bay pharmaceutical concentrations range between 0.04–651.83 ng/g, 0.31–342.09 ng/g, 0.04–502.40 ng/g and 0.08–686.84 ng/g, respectively (Fig. 2). Whereas in the wet season, the concentrations ranged between 0.02–1632.61 ng/g (Tudor creek), 0.05–530.71 ng/g (Makupa creek), 0.02–723.56 ng/g (Mtwapa creek) and 0.17–36.25 ng/g (Gazi Bay). The highest concentration of pharmaceuticals was observed in Tudor creek during both the wet and dry seasons, with a range of 0.04–651.8 ng/g and 0.01–2580.6 ng/g, respectively. These concentrations were higher than those reported in False Bay, South Africa, 92.08–171.89 ng/g (Ojemaye and Petrik 2021); Mediterranean coastal lagoon, Spain, BDL–54.2 ng/g (Moreno-González et al. 2015); Bohai Bay, China, 7.71–130.36 ng/g (Cheng et al. 2016); Augusta Bay, southern Italy, 9–26 ng/g (Feo et al. 2020); Pego–Oliva Marshlands, Valencia, Spain, < 30 ng/g (Lara-Martín et al. 2014); Jiaozhou Bay, north China, 3.62–21.4 ng/g (Peng et al. 2020); and in Todos of Santos Bay, Salvador Bahia, Brazil, average 39 ng/g (Beretta et al. 2014). The higher concentrations indicate untreated sewage discharge into Tudor, Makupa, Mtwapa creeks and Gazi Bay.

**Fig. 3:**
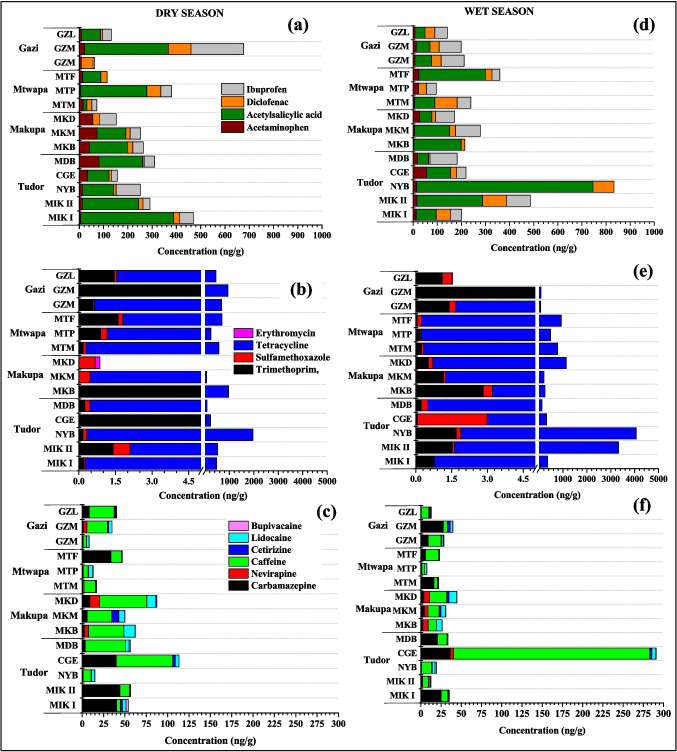
Concentration of pharmaceutical in surface sediment (ng/g dry weight) of Tudor (FJE, MDB, CGE, NYB, MIKII, and MIKI), Makupa (MKM, MKB, and MKD and Mtwapa creek (MTF, MTP, and MTM), and Gazi Bay (GZO, GZL, and GZM) during dry (panels (**a**), (**b**), and (**c**)) and wet seasons (panels (**d**), (**e**), and (**f**). Note there is a non-linear break included on the concentration scale for panels (**b**), and (**e**) due to the large differences in concentration

Even though analgesics (acetaminophen and acetylsalicylic acid) and non-steroidal anti-inflammatory drugs (NSAIDs) (diclofenac and ibuprofen) are highly biodegradable, acetaminophen, diclofenac and ibuprofen were still detected in the surface sediment of Makupa, Mtwapa, Tudor creeks and Gazi Bay during both the wet and dry seasons, at concentrations ranging from 11.42 to 202.52 ng/g and 10.48 to 362.38 ng/g, respectively. The NSAID acetaminophen concentration was high at the MDB site (dry season) and CGE site (wet season) in Tudor creek, with a concentration of 80.79 ng/g and 55.96 ng/g, respectively (Fig. 3a). These values were similar to those reported in False Bay, South Africa (Ojemaye and Petrik 2021), but higher than the concentration in Pego–Oliva Marshlands, Eastern Spain (Vazquez-Roig et al. 2012). Ibuprofen concentration was high at the GZO site (within Gazi Bay) during the dry season, with a concentration of 216.45 ng/g and MDB (112.42 ng/g) within Tudor creek in the wet season. These values were similar to those reported in Golden Horn Estuary, Sea of Marmara, Turkey (Korkmaz et al. 2022) but higher than in Todos os Santos Bay, Brazil (Beretta et al. 2014).

Similarly, the highest concentration of diclofenac was observed in GZO (in Gazi Bay), with a concentration of 93.41 ng/g in the dry season and MIKII (100.08 ng/g) in Tudor creek during the wet season. Similar concentrations (Table 2) were reported in False Bay, South Africa (Ojemaye and Petrik 2021) and Golden Horn Estuary, Sea of Marmara, Turkey (Korkmaz et al. 2022), but lower than the value investigated in Todos os Santos Bay, Brazil (Beretta et al. 2014). On the other hand, acetylsalicylic acid concentration in the wet season was high in NYB within Tudor creek (731.53 ng/g) and GZO in Gazi Bay with a concentration of 345.24 ng/g during the wet season. The high concentration of ibuprofen, diclofenac and acetylsalicylic acid in Gazi Bay could result from non-point source pollution in the Bay from longshore transport from Msambweni hospital. The high concentration of these compounds detected in surface sediment suggests continuous contamination from domestic sewage effluent into marine aquatic environment. It is worth noting that acidic pharmaceuticals like acetylsalicylic acid, diclofenac and ibuprofen have pKa values which range from 3.5 to 4. Hence, high sorption to sediment is expected in combination with electrostatic attraction between positively charged mineral surfaces (e.g., Al and Fe oxides) and negatively charged pharmaceuticals (Korkmaz et al. 2022; Krascsenits et al. 2008).

**Table 2:** Concentrations of pharmaceuticals in surface sediments in other sites worldwide

Pharmaceutical	Site	Country	Concentration (ng/g)	References
Acetaminophen	False Bay	South Africa	34.28–67.92	Ojemaye and Petrik (2021)
Acetaminophen	Pego–Oliva Marshlands	Eastern Spain	2.4 ± 3.9	Vazquez-Roig et al. (2012)
Ibuprofen	Todos os Santos Bay	Brazil	0.77–15.6	Beretta et al. (2014)
Ibuprofen	Golden Horn Estuary, Sea of Marmara,	Turkey	< 91–215	Korkmaz et al. (2022)
Diclofenac	False Bay	South Africa	92.08–171.89	Ojemaye and Petrik (2021)
Diclofenac	Todos os Santos Bay	Brazil	< 0.01–1.06	Beretta et al. (2014)
Diclofenac	Golden Horn Estuary, Sea of Marmara	Turkey	< 30.6	Korkmaz et al. (2022)
Caffeine	San Francisco Bay	USA	< RL–29.7	Klosterhaus et al. (2013)
Caffeine	Santos Bay (São Paulo)	Brazil	23.4	Pereira et al. (2016)
Caffeine	Todos os Santos Bay	Brazil	0.28–23.4	Beretta et al. (2014)
Tetracycline	Bohai Bay,	China	nd–94.79	Cheng et al. (2016)
Tetracycline	Gulf of Finland	Russia	0.5–0.7	Chernova et al. (2021)
Tetracycline	Dalian,	China	1.66–1.74	Na et al. (2013)
Tetracycline	Zhuhai City	China	nd–25.5	Li et al. (2018)
Tetracycline	Jiaozhou Bay	China	nd–3.45	Peng et al. (2020)
Tetracycline	Beibu Gulf	China	3.24–14.08	Leng et al. (2020)
Sulfamethoxazole	Beibu Gulf,	China	nd–2.87	Leng et al. (2020)
Sulfamethoxazole	Baltic Sea	Baltic Sea	nd–419.2	Siedlewicz et al. (2016)
Sulfamethoxazole	San Francisco Bay	USA	< RL–0.7	Klosterhaus et al. (2013)
Sulfamethoxazole	Yangtze Estuary, coastal	China	nd–100	Shi et al. (2014a)
Sulfamethoxazole	Bohai Bay,	China	nd–3.95	Cheng et al. (2016)
Sulfamethoxazole	Pego–Oliva Marshlands	Spain	0.1 ± 0.3	Vazquez-Roig et al. (2012)
Trimethoprim	Gulf of Finland	Russia	0.1–0.2	Chernova et al. (2021)
Trimethoprim	San Francisco Bay	USA	< RL–18.2	Klosterhaus et al. (2013)
Trimethoprim	Baltic Sea	Baltic Sea	nd–2.46	Siedlewicz et al. (2016)
Trimethoprim	Mediterranean coastal lagoon	Spain	bql–0.8	Moreno-González et al. (2015)
Trimethoprim	Polish coastal zone	Baltic Sea	< MQL to 35.7	Siedlewicz et al. (2016)
Erythromycin	Todos os Santos Bay	Brazil	< 0.01–2.29	Beretta et al. (2014)
Erythromycin	San Francisco Bay	USA	< RL–3.4	Klosterhaus et al. (2013)
Carbamazepine	Todos os Santos Bay	Brazil	< 0.01–4.48	Beretta et al. (2014)
Carbamazepine	False Bay	South Africa	33.27–67.92	Ojemaye and Petrik (2021)
Carbamazepine	Gulf of Finland	Russia	0.5–62.2	Chernova et al. (2021)
Carbamazepine	Golden Horn Estuary, Sea of Marmara,	Turkey	< 32–118	Korkmaz et al. (2022)
Carbamazepine	Pego–Oliva Marshlands	Eastern Spain	0.9 ± 0.5	Vazquez-Roig et al. (2012)

< *RL* concentrations less than the reporting limit, *nd* not detected, *MQL* method quantitation limit, *bql* below the limit of quantification

The target antibiotics (erythromycin, tetracycline, trimethoprim and sulfamethoxazole) were detected in Makupa, Mtwapa and Tudor creek stations and Gazi Bay. The concentrations of the four antibiotics showed the following order tetracycline > trimethoprim > sulfamethoxazole > erythromycin. Gazi Bay had the highest concentration of antibiotics during the dry season, with a sum concentration of ∑690 ng/g, and in the wet season, Tudor creek had the highest concentration, with a sum concentration of ∑2582 ng/g. Tetracycline concentrations in dry and wet seasons ranged between DL–1969 ng/g and DL–4064 ng/g, respectively, with the highest concentration observed in NYB within Tudor creek (Fig. 3b). These values were considerably higher than those (see Table 2) reported in Bohai Bay, China (Cheng et al. 2016), Gulf of Finland (Russia) (Chernova et al. 2021), Zhuhai City, China (Li et al. 2018), Dalian, China (Na et al. 2013) and Beibu Gulf, China (Leng et al. 2020). The high concentration of tetracycline may be due to its low price and high efficiency in treating diseases in both humans and animals, as well as its use as a growth promotor in aquaculture and livestock (Li et al. 2023; Shi et al. 2014b). In addition, tetracycline easily adsorbs onto sediments and particulates, as well as degrades relatively quickly in the environment. However, the long-term and continuous discharge of large quantities into receiving waters leads to its ubiquitous presence and high concentration (Guo et al. 2019; Vazquez-Roig et al. 2012). Sulfamethoxazole and trimethoprim are often used in combination to treat various bacterial infections (Bagnis et al. 2020). The concentration of sulfamethoxazole in the dry season ranged between 0.05–0.68 ng/g, 0.45–2.59 ng/g and 0.11–0.26 ng/g in Tudor, Makupa and Mtwapa creeks, respectively. Whereas in the wet season, the concentration ranged from DL–2.91 ng/g (Tudor creek), 0.08–0.38 ng/g (Makupa creek) and 0.03–0.17 ng/g (Mtwapa creek). High sulfamethoxazole concentration was reported in MKB (Makupa creek) in the dry season and CGE (Tudor creek) in wet season with a concentration of 2.59 ng/g and 2.91 ng/g, respectively. These concentrations were comparable to those (see Table 2) in San Francisco Bay, USA (Klosterhaus et al. 2013); Pego–Oliva Marshlands, Spain (Vazquez-Roig et al. 2012); Beibu Gulf, China (Leng et al. 2020); Baltic Sea (Siedlewicz et al. 2016); and Bohai Bay, China (Cheng et al. 2016). It is worth mentioning that sulfamethoxazole is the most frequently detected sulfonamide because it does not degrade easily and has a half-life of 85 to > 100 days (Siedlewicz et al. 2018). On the other hand, trimethoprim concentration during the dry season in Tudor, Makupa and Mtwapa creeks ranged between 0.17–26.97 ng/g, DL–6.89 ng/g and 0.18–1.61 ng/g, respectively, with the highest concentration observed in CGE (26.97 ng/g) in Tudor creek. Whereas in the wet season, the concentration of trimethoprim ranged between 0.07–1.53 ng/g, 0.53–2.83 ng/g and 0.05–0.24 ng/g in Tudor, Makupa and Mtwapa creeks, respectively. These values were consistent with those reported (see Table 2) in Gulf of Finland, Russia (Chernova et al. 2021); Baltic Sea (Siedlewicz et al. 2016); and the Mediterranean coastal lagoon, SE Spain (Moreno-González et al. 2015); San Francisco Bay, USA (Klosterhaus et al. 2013); and Polish coastal zone, Baltic Sea (Siedlewicz et al. 2018). Among the four target antibiotics in this study, erythromycin showed the lowest concentration with a sum of ∑1.48 ng/g and ∑ 0.48 ng/g in the dry and wet seasons, respectively (Fig. 3b). MKB in Makupa creek had the highest concentration of erythromycin with a concentration of 0.69 ng/g (dry season) and 0.08 ng/g (wet season). Similar concentrations were reported (see Table 2) in Todos os Santos Bay, Brazil (Beretta et al. 2014), and San Francisco Bay, USA (Klosterhaus et al. 2013). Antibiotics are one of the most commonly used classes of pharmaceuticals in hospitals, households and veterinary medicine to prevent and treat bacterial infections in humans and animals and/or as growth promoters in animal agriculture and aquaculture industry (Peng et al. 2020; Sanusi et al. 2023). They are of concern since their accumulation in the sediments could either cause adverse toxic effects on the non-target organisms or stimulate antibiotic resistance genes among microorganism communities that may pose health threats to humans, animals and resultant ecological risk (Liang et al. 2013).

Antihistamine drug caffeine concentration ranged between 3.83–65.82 ng/g and 0.43–242.80 ng/g during dry and wet seasons, respectively. Tudor creek had the highest concentrations of caffeine, particularly in CGE, during both dry and wet seasons, with a concentration of 65.82 ng/g and 242.80 ng/g, respectively (Fig. 3c). These values were higher than the findings (Table 2) in Todos os Santos Bay, Brazil (Beretta et al. 2014); San Francisco Bay, USA (Klosterhaus et al. 2013); and Santos Bay (São Paulo), Brazil (Pereira et al. 2016). Caffeine was detected in all the study sites, most probably caused by the fact that approximately 5% of ingested caffeine is excreted in the urine and eventually reaches the aquatic environment through sewage systems as well as through the disposal of food, beverages, tobacco and medicines containing caffeine (Paíga and Delerue-Matos 2017; Rigueto et al. 2020). In addition, caffeine has a half-life of between 3.5 days and more than 100 days in estuarine and coastal waters (Chernova et al. 2021; Nödler et al. 2014). Cetirizine was detected at all of the sample sites, with the highest concentration detected at the MKM site (8.33 ng/g) in Makupa creek during the dry season and at the GOF site (3.27 ng/g) in Gazi Bay during the wet season (Fig. 3c). High concentration could be attributed to the fact that 70% of the cetirizine dose (maximum of 20 mg/tablet/day) is excreted unchanged by renal mechanisms, and approximately 10% in the faeces and 8 to 10% is metabolised by the P450 cytochrome oxidase pathway (Almeida et al. 2017; Campoli-Richards et al. 1990).

Bupivacaine and lidocaine are widely used local analgesic anaesthetics that enhance postoperative pain control and reduce the need for narcotics after surgery (Collins et al. 2013). During the dry season, Tudor creek exhibited the highest concentration of bupivacaine in the MIKI site at 2.62 ng/g, while in the wet season, the NYB site had a concentration of 1.84 ng/g. In contrast, lidocaine concentrations peaked at Makupa creek, with 13.21 ng/g recorded at the MKB site during the dry season and 9.75 ng/g at the MKD site in the wet season.

Antiretroviral drug, nevirapine, ranged from DL to 11.16 ng/g and DL to 7.06 ng/g in the dry and wet seasons, respectively. The highest concentration of nevirapine in the dry and wet seasons was observed in station MKD (within Makupa creek), with a concentration of 11.16 ng/g and 7.06 ng/g, respectively. The observed presence of nevirapine concentration in the sampled areas could be due to its wide use for the treatment of HIV and for the prevention of mother to child transmission (Schoeman et al. 2017).

Antiepileptic drugs carbamazepine concentration in the dry and wet seasons showed a sum concentration of ∑191.32 ng/g and ∑151.45 ng/g, respectively. Carbamazepine concentration in the surface sediment was high in Tudor Creek, with a mean concentration of 25.64 ± 0.29 ng/g and 16.63 ± 0.55 ng/g in the dry and wet seasons, respectively. Stations MIKII (44.18 ng/g) and CGE (36.31 ng/g) in Tudor Creek were observed to have the highest concentration of carbamazepine during the dry and wet seasons, respectively (Fig. 3c). These results showed similar concentrations with previous studies (Table 2) in False Bay, South Africa (Ojemaye and Petrik 2021); Golden Horn Estuary, Sea of Marmara, Turkey (Korkmaz et al. 2022); and Gulf of Finland, Russia (Chernova et al. 2021) but higher than the values reported in Pego–Oliva Marshland, Spain (Vazquez-Roig et al. 2012), and Todos os Santos Bay, Brazil (Beretta et al. 2014). Carbamazepine has a pKa of 13.9 and has no charge in the pH range of 7.8–8.5, and this is probably why it has a low affinity to sorb onto sediment particulates. Hence, low concentrations were observed despite the continuous discharge of raw domestic sewage into the creeks, and with a mean half-life ~ 82 days, carbamazepine could persist within receiving environments (de Wilt et al. 2018; Korkmaz et al. 2022).

### Influence of environmental factors on pharmaceutical

The distribution and sorption of pharmaceuticals in sediments are affected by several factors, such as sediment type, electric conductivity, calcium carbonate, total organic carbon (TOC), salinity and pH (Peng et al. 2020; Sadutto et al. 2021). As well as the physicochemical properties of pharmaceuticals (like, octanol–water partition coefficient [log Kow], dissociation constant [pKa]) (Korkmaz et al. 2022), which may be exacerbated by differences between samples collected along different sites during different seasons, as occurred in this study. Sediment particle distribution in various stations in Makupa, Mtwapa, Tudor and Gazi is illustrated in Table 3. A high sand content was observed in Gazi Bay in the dry and wet seasons, with a mean percentage of 62.7% and 82.8%, respectively. Mtwapa creeks had the highest percentage of clay content ranging from 17%–33% and 8%–50% in the dry and wet seasons, respectively. This could be due to the seasonal river (River Mto Mkuu), which flows into the creek, discharging finer sediment (Okuku et al. 2020). In comparison, silt content was high in Makupa creek, with a percentage content of 85.4% and 58.6% in the dry and wet seasons, respectively. Despite high clay content in Mtwapa creek in the dry and wet seasons, a strong insignificant correlation between clay content and total pharmaceutical concentration (*r* = 0.953) was observed only in the wet season (Figure S1). Meanwhile, in Makupa creek, clay content and total pharmaceuticals showed a strong correlation with no significant difference between the dry season (*r*^2^ = 0.993, *p* > 0.05) and wet season (*r* =  − 0.834, *p* > 0.05). Pharmaceuticals (ibuprofen, tetracycline, erythromycin, bupivacaine trimethoprim and sulfamethoxazole) strongly but insignificantly correlated with percentage clay content in Makupa, Mtwapa creek and Gazi Bay. Similarly, a strong correlation between the percentage clay content with acetaminophen, acetylsalicylic acid and diclofenac was observed in Tudor and Makupa creeks. This indicates that the higher the percentage of clay content, the higher the pharmaceutical concentration. This observation could be attributed to the fact that a smaller sediment grain size (< 0.063 mm) has a larger surface area and high cation exchange capacity, which tends to have a complex active sorption site available, resulting in increased pharmaceutical enrichment (Liang et al. 2013; Peng et al. 2020).

**Table 3:** Physicochemical characteristics of surface sediment samples collected from Makupa, Mtwapa, Tudor creeks and Gazi Bay

Creeks/Bay	Station	Dry season					Wet season				
		TOC (%)	Organic matter (%)	Particle size distribution			TOC (%)	Organic matter (%)	Particle size distribution		
				Sand (%)	Silt (%)	Clay (%)			Sand (%)	Silt (%)	Clay (%)
Tudor	MIK	0.36	4.8	24.3	68.6	7.2	0.33	21.8	33.4	56.9	9.7
	NYB	0.44	5.7	25.6	72.4	2.1	0.26	12.5	36.3	51.3	12.5
	CGE	0.28	4.6	47.6	51.5	1.0	0.58	26.0	69.6	30.0	0.4
	MDB	0.06	1.3	71.3	28.7	0.0	0.02	2.2	98.7	1.3	0.0
Makupa	MKB	0.45	5.4	0.0	92.4	7.6	0.34	13.5	19.9	62.7	17.5
	MKM	0.47	5.5	17.9	81.9	0.2	0.29	8.8	27.4	64.0	8.6
	MKD	0.78	7.3	17.9	81.9	0.2	0.57	22.8	48.9	49.0	2.1
Mtwapa	MTM	0.27	4.3	0.0	67.0	33.0	0.18	9.4	42.9	34.0	23.1
	MTP	0.31	5.8	6.5	76.6	16.9	0.30	9.1	18.9	30.8	50.3
	MTF	0.23	5.2	0.2	74.4	25.4	0.18	29.0	23.0	69.5	7.5
Gazi	GZO	0.65	7.4	48.1	51.9	0.0	1.19	40.6	70.7	29.2	0.1
	GZL	0.60	7.5	97.8	2.2	0.0	0.10	5.1	83.6	16.4	0.0
	GZM	0.00	0.7	42.2	57.8	0.0	0.04	3.8	94.0	6.0	0.0

On the other hand, the percentage of organic matter in the surface sediment was high in MKD (in Makupa creek), with 7.3% and 22.8% in dry and wet seasons, respectively, and this could be due to the presence of large amounts of waste compounds and relatively high microorganism activities since the area was used as a dump site for solid waste in previous years. In Gazi Bay, the percentage of organic matter was high in GZO during dry and wet seasons measuring 7.49% and 40.6%, respectively, which may be attributed to seagrass, mangrove leaves and seaweed decay. Organic matter provides the cation-exchange capacity in sediment that reacts with pharmaceutical charged sites (Liang et al. 2013). In Gazi Bay, all pharmaceuticals except caffeine strongly correlated with organic matter. On the other hand, acetylsalicylic acid, nevirapine and caffeine strongly correlated with organic matter in Makupa creek. Mtwapa creek also strongly correlated with organic matter and tetracycline, carbamazepine, nevirapine, caffeine and lidocaine (Figure S1). Artifon et al. (2019) observed that the origin of different organic matter (e.g. autochthonous or allochthonous), the form (whether it is dissolved, particulate or colloidal) and composition (non-humic or humic substances) can greatly affect the absorption/desorption equilibrium of contaminants.

The percentage of total organic carbon (%TOC) in surface sediments was higher in the dry season than in the wet season (Table 3). A high TOC content during dry and wet seasons was observed in MKD (dry season, 0.78%; wet seasons 0.57%) in Makupa creek, MTP (dry season, 0.31%; wet seasons 0.30%) in Mtwapa creek and GZO (dry season, 0.65%; wet seasons 1.19%) in Gazi Bay. At the same time, Tudor creek reported a high %TOC in NYB in the dry season (0.44%) and CGE (0.58%) in the wet season. Marine sediment layers with an organic carbon content (*C*_org_) > 2% are defined as sapropels (organic-rich layers), while sediment layers with a (*C*_org_) content of 0.5%–2% are called sapropelic (organic-poor sediments) (Nijenhuis 1999; Shaw and Evans 1984). This shows that surface sediment from Tudor, Makupa, Mtwapa creek and Gazi Bay have a sapropelic layer with low organic content. It is worth mentioning that pharmaceuticals tend to have a high adsorption capacity to sediment with high organic carbon content (Liang et al. 2013; Rimayi et al. 2018; Siedlewicz et al. 2016). Hence, in this study, a strong negative correlation between TOC content and pharmaceutical concentration in the dry and wet seasons in the surface sediments was observed in Mtwapa creek (dry season; *r* =  − 0.903, *p* > 0.05; wet season; *r* = 0.773, *p* > 0.05). Conversely, TOC content and pharmaceutical concentration strongly correlated in Tudor creek (*r* = 0.707) during the dry season and Makupa creek (*r* = 0.984) during the wet season. This highlights that the different pharmaceuticals that interact with the sediments have very different adsorption mechanisms. Organic compounds, as measured with TOC, are integral in predicting the sorptive capacity of organic contaminants in sediment. However, a range of environmental parameters, such as biological activity, pH, salinity and the local hydrodynamics that are typically observed in coastal systems, may alter this prediction (Castro 2019; Sadutto et al. 2021).

### Seasonal variation of pharmaceuticals in sediment

The sorption mechanism of pharmaceuticals to sediment may include interactions with minerals containing positive charges. Sediment can contain aluminosilicate minerals such as feldspar, biotite and muscovite and contain iron oxides and aluminium oxides, which can have positively charged surfaces (Krascsenits et al. 2008; Lorphensri et al. 2007). Low concentrations of these pharmaceuticals were detected during both the dry and wet seasons, except for acetylsalicylic acid, diclofenac, ibuprofen, caffeine and tetracycline (Fig. 3) in Tudor, Makupa, Mtwapa creek and Gazi Bay. Pharmaceutical concentrations in the surface sediment of Tudor, Makupa, Mtwapa creek and Gazi Bay showed no significant difference between the wet and dry seasons (pair-sample *t*-test, *p* > 0.05). This might be attributed to a combination of factors such as seawater dilution effect from storm water, low dilution during flooding and ebbing of the creeks and slow degradation due to continuous discharge of domestic sewages into the creeks which could change the ocean pH (Wanjeri et al. 2023). These changes in pH will likely affect any of the local carbon cycles and in turn likely lead to changes in the solubility of acidic and basic substances. The cumulative effect of these changes will impact how these compounds ionise and result in changes to their lipophilic characteristics, such as enhancement of their lipophilicity (Dionísio et al. 2020). Pharmaceuticals detected in the marine environments of Tudor, Makupa, Mtwapa creek and Gazi Bay during both wet and dry seasons are associated with sewage effluent, wastewater from poorly maintained treatment plants and unregulated discharges (from informal settlements) into the creeks due to increased population and industrialization.

### Occurrence of pharmaceutical residue in macroalgae

**Fig. 4:**
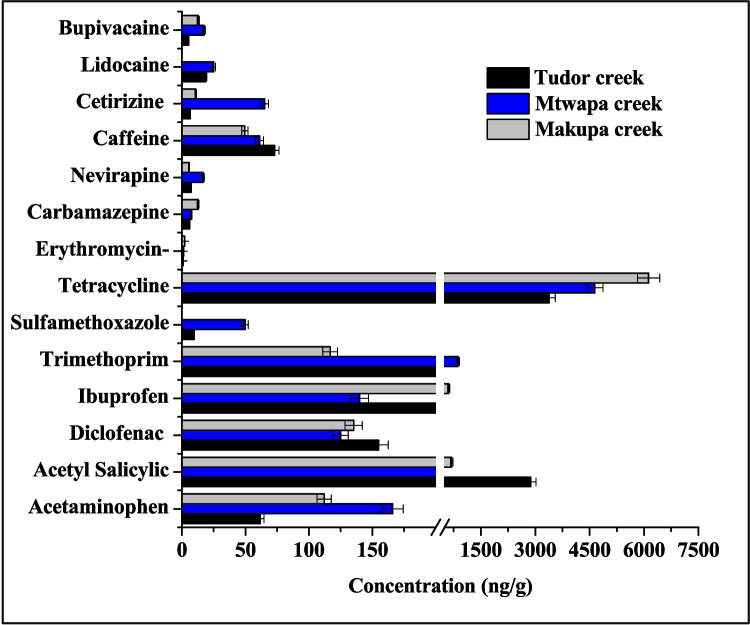
Mean concentration (± SE) of pharmaceuticals in macroalgae (ng/g dry weight) from Tudor, Makupa and Mtwapa creek

The mean concentration of pharmaceuticals in seven different types of ulvophyceae species (*Cladophora sudanensis*, *Chaetomorpha crassa*, *Chaetomorpha indica*, *Enteromorpha kylinii*, *Ulva reticulate*, *Ulva lactuca* and *Cladophora sibugae*) in Makupa, Mtwapa and Tudor creeks are illustrated in Fig. 4. The concentration of pharmaceuticals in different species of ulvophyceae in Tudor, Mtwapa and Makupa creeks ranged between 0.86–3383 ng/g, 1.24–4637 ng/g and 2.14–6123 ng/g, respectively, indicating the accumulation capacity of algae with tetracycline showing the highest concentration (Fig. 4).

The concentration of pharmaceuticals in our study was higher than the values reported in the Saudi Red Sea (not detected (nd)–44.3 ng/g) (Ali et al. 2018). Tudor creek macroalgae had a high concentration of acetylsalicylic acid, carbamazepine, caffeine and lidocaine, whereas Mtwapa creek algae had a high concentration of acetaminophen, trimethoprim, erythromycin, nevirapine, cetirizine and bupivacaine (Fig. 4). Makupa creek algae had a high concentration of diclofenac, ibuprofen and tetracycline.

Acetaminophen concentration in macroalgae in this study ranged between DL–429.48 ng/g, which were higher than values reported in False Bay, South Africa, and 122.97–129.69 ng/g (Ojemaye and Petrik 2021). On the other hand, diclofenac concentration across the stations ranged from 1.09 to 245.54 ng/g, which was within the range values with False Bay, South Africa, 153.94–205.31 ng/g (Ojemaye and Petrik 2021), whereas the concentration of acetylsalicylic acid in macroalgae of Tudor, Mtwapa and Makupa creek had a sum concentration of 2874.91 ng/g, 424.92 ng/g and 693.81 ng/g, respectively.

**Fig. 5:**
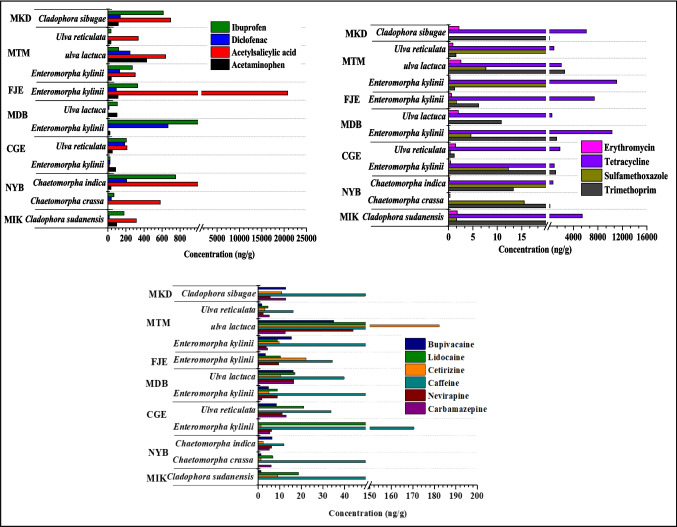
Concentration of pharmaceuticals in different macroalgae, species (*Cladophora sudanensis, Chaetomorpha crassa*, *Chaetomorpha indica*, *Enteromorpha kylinii*, *Ulva reticulate*, *Ulva lactuca* and *Cladophora sibugae*) in Tudor, Makupa and Mtwapa creeks

Among the antibiotics studied, tetracycline showed the highest concentration in all macroalgae species (1.24–1076 ng/g). Sulfamethoxazole concentration (DL–84.64 ng/g) was lower than values reported in False Bay, South Africa, 21.93–175.76 ng/g (Ojemaye and Petrik 2021). Antiepileptic, carbamazepine concentration in macroalgae ranged between 0.06–16.40 ng/g, which were also lower than values in False Bay, South Africa; 34.24–58.65 ng/g (Ojemaye and Petrik 2021) but higher than values in Saudi Red Sea; and nd–1.7 ng/g (Ali et al. 2018). For caffeine, the concentration ranged between 12.04 and 170.48 ng/g. These values were higher than those investigated in Saudi Red Sea, nd–44.3 ng/g (Ali et al. 2018). High concentrations of acetylsalicylic acid (20,787 ng/g), caffeine (170.48 ng/g), ibuprofen (1076 ng/g), lidocaine (65.38 ng/g) and tetracycline (11,059 ng/g) were detected in *Enteromorpha kylinii* from stations FJE, MDB, CGE and MTM, respectively (Fig. 5). On the other hand, *ulva lactuca* found in MTM within Mtwapa creek had the highest concentration of acetaminophen (49.67 ng/g), diclofenac (245.54 ng/g), trimethoprim (2605.91 ng/g), sulfamethoxazole (84.64 ng/g), erythromycin (2.52 ng/g), nevirapine (44.02 ng/g) and cetirizine (170.48 ng/g). The accumulation of pharmaceuticals in the different species of macroalgae samples varied significantly (*F* = 2.053, *p* < 0.05). This could be due to the variations in their uptake of chemicals and the availability of these chemicals in seawater and sediment. In addition, the compounds’ uptake processes and physiochemical properties in combination with the physiology of macroalgae are expected to play an important role in the complex interaction of pollutant bioaccumulation and transformation (Ali et al. 2018).

### Bioconcentration factor (BCF)

**Fig. 6:**
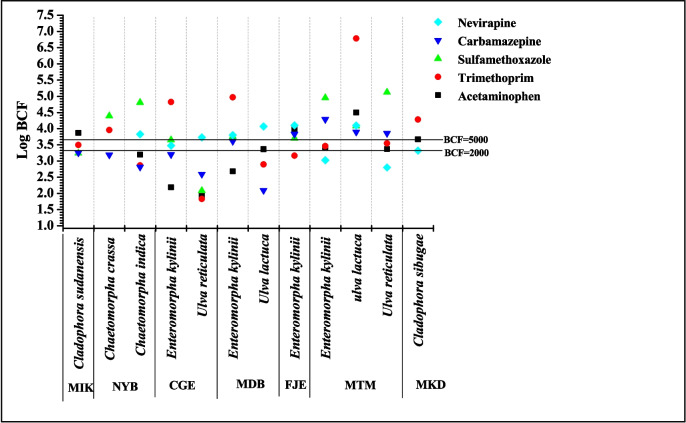
Summary of log BCF values for pharmaceuticals in different macroalgae species in dry weight

The BCF of different macroalgae species at various stations in Tudor, Makupa and Mtwapa creek were calculated and are illustrated in Fig. 6. At the NYB site, the macroalgae *Chaetomorpha crassa* had two pharmaceutical compounds (trimethoprim, sulfamethoxazole) with BCF values higher than 5000 L/kg (Log 3.7). At the same site, another macroalgae sample, *Chaetomorpha indica*, also had two pharmaceutical compounds (sulfamethoxazole and nevirapine) with BCF values higher than 5000 L/kg (Log 3.7). While at the MDB and FJE sites, *Enteromorpha kylinii* had 4 compounds (trimethoprim, sulfamethoxazole, carbamazepine and nevirapine) with BCF values higher than 5000 L/kg (Log 3.7). Similarly, *Ulva lactuca* (trimethoprim, sulfamethoxazole, carbamazepine and nevirapine) and *Ulva reticulata* (sulfamethoxazole and carbamazepine) in the MTM site also exhibited BCF values higher than 5000 L/kg for certain pharmaceutical compounds. This indicates that trimethoprim, sulfamethoxazole, carbamazepine and nevirapine have the potential to bioaccumulate in marine macroalgae. The high bioaccumulation of pharmaceuticals in macroalgae can be influenced by relatively low wave action and continuous direct sewage discharge from homes and other domestic buildings and this explains their high concentration in Tudor, Makupa and Mtwapa creeks.

### Comparison of pharmaceutical concentration in sediment and macroalgae

**Fig. 7:**
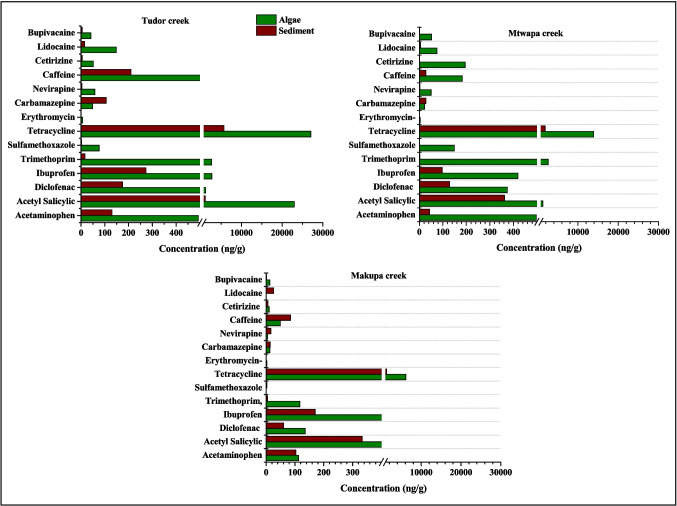
Sum concentration of pharmaceuticals in sediment and macroalgae in Tudor, Makupa and Mtwapa creeks

Pharmaceutical concentrations between sediment and macroalgae showed no significant difference (pair-sample *t*-test, *p* > 0.05) in Tudor, Makupa and Mtwapa creeks. This is because macroalgae tend to settle directly onto the sediment substrate, thus in contact with this contamination sink. The concentration of pharmaceuticals was observed to be higher in macroalgae compared to the sediment of Tudor, Makupa and Mtwapa creeks (Fig. 7). This could be attributed to environmental factors such as light exposure, nutrient availability and water temperature, which can influence the growth and metabolic activity of algae. These factors may enhance the uptake of pharmaceuticals during periods of active growth (Khan and Barros 2023). Additionally, variations in the bioadsorption of pharmaceuticals on the microalgal cell wall or onto extracellular polymeric substances, as well as the availability of these chemicals in seawater and sediment, play a significant role (Maryjoseph and Ketheesan 2020; Ojemaye and Petrik 2021). Furthermore, macroalgae possess a larger surface area relative to their volume, along with the presence of polysaccharides and other organic compounds that improve the adsorption of both hydrophilic and hydrophobic pharmaceuticals (Carafa et al. 2007).

Tetracycline showed the highest concentration in sediment and macroalgae in all the creeks compared to other pharmaceutical compounds (Fig. 7). This may be attributed to the relative stability of tetracycline in low-oxygen or anaerobic sediment environments and the continuous discharge of large quantities into the creeks, where it can persist for long periods without significant degradation (Cetecioglu et al. 2013). Tetracycline is moderately hydrophilic and highly ionizable, depending on the pH, which allows it to exist in multiple forms (neutral, cationic and anionic). This variability enhances its ability to interact with various sediment particles, particularly those containing organic matter or clay minerals (Wang et al. 2021). Additionally, tetracycline has a strong affinity for cations such as calcium and magnesium, enabling it to form complexes with these ions commonly found in sediments (Carlotti et al. 2012). The low concentration of erythromycin observed in sediment and macroalgae in all the creeks (Fig. 6) can be attributed to its high water solubility, susceptibility to degradation (from light and microbial processes) and its limited affinity for sediment particles and algal tissues (Schafhauser et al. 2018).

### Potential environmental risk assessment

The potential ecological risks of pharmaceuticals in the marine aquatic environment have raised public concerns. Hence, the mean measured concentration of pharmaceuticals detected in sediments was assessed by computing the risk quotient (RQ) for algae, invertebrates and fish. The data and results of risk assessment are described in detail in Table S1. Acetaminophen and trimethoprim concentrations posed a high risk (Log10 RQ > 0) to fish, crustacea and algae, while sulfamethoxazole posed a high risk to algae and nevirapine to fish in Tudor, Makupa, Mtwapa creek and Gazi Bay during both dry and wet seasons, except for fish and crustacea in Mtwapa creek during the wet season. The concentration of carbamazepine and sulfamethoxazole posed a medium risk (− 1 < Log10 RQ < 0) to fish and crustacea of Tudor, Makupa and Mtwapa creeks and Gazi Bay. The pharmaceuticals (acetaminophen, sulfamethoxazole, trimethoprim, carbamazepine and nevirapine) posed a medium to a higher risk to algae, invertebrates and fish in the surface sediment during the dry and wet seasons due to the continuous discharge of domestic and industrial sewage and wastewater into the creeks. This was observed in the high bioaccumulation of pharmaceuticals in the different species of macroalgae in the Tudor, Makupa and Mtwapa creeks (Fig. 6). Since algae are the primary producers in the marine environment, and are the major contributors and important producers of carbon sinks in the marine ecosystem, invertebrates are a significant part of the bottom of the food chain. Changes in their population structure and quantity of algae and invertebrates will have a major influence on the ecosystem in Mombasa peri-urban creeks and Gazi Bay (Peng et al. 2019, 2020). The environmental risk assessment in this study focused solely on the ecotoxicity that individual pharmaceuticals may present to aquatic organisms. However, pharmaceuticals are found in aquatic environments as complex mixtures of various therapeutic groups, and the toxicity values obtained here do not capture the overall toxicity in sediment.

## Conclusions

The distribution of pharmaceuticals in the surface sediment and macroalgae during different seasons was investigated for the first time in Makupa, Mtwapa, Tudor creeks and Gazi Bay (reference environment). Pharmaceuticals were detected in most of the sampling stations at Makupa, Mtwapa and Tudor creeks. This could be related to substantial outflows of fresh untreated domestic and municipal raw sewage water either from a point or non-point source due to high urbanization and population density on the coast. In addition, there is no sewage treatment plant (STP) along the creeks, and most wastewater is discharged directly into the marine environment.

Tudor creek exhibited the highest concentration of pharmaceuticals during both the wet and dry seasons compared to Makupa and Mtwapa creeks. This may be attributed to multiple point sources of fresh sewage effluent and a possibly poorly maintained sewage treatment plant associated with a level 5 hospital. Despite being used as a reference environment, the concentration of pharmaceuticals was detected in the surface sediment of Gazi Bay, and this could be due to the non-point source pollution in the Bay from longshore transport from Msambweni hospital as well as input from seasonal rivers. The concentration of pharmaceuticals in surface sediment during dry and wet seasons showed no season variations. This confirms that sediments can act as a sink for pharmaceuticals in the aquatic environment and a potential source of secondary pollution with changes in environmental conditions. Pharmaceuticals posed a medium to higher risk to algae, invertebrates and fish in the surface sediment during the dry and wet seasons. Notably, a high concentration of pharmaceuticals was detected in macroalgae compared to surface sediment in Makupa, Mtwapa and Tudor creeks. The presence of these pharmaceuticals in macroalgae shows that macroalgae can bioaccumulate pharmaceuticals from the surrounding environment.

Therefore, the present study complements and confirms the impact of sewage on the marine environment of Mombasa peri-urban creek. These findings, together with the detection of pharmaceuticals in Mombasa peri-urban creek sediment, require further research to understand the distribution, transformation, fate and impacts of pharmaceuticals in the aquatic environment. In addition, there is a need to sensitise the residents of coastal cities on the impact of sewage effluent in the marine environment and enact strict measures to limit the discharge of sewage effluents containing these contaminants into the marine environment.

## Supplementary Information

Below is the link to the electronic supplementary material.

Supplementary file1 (DOCX 970 KB)

## Data Availability

The authors declare that the data supporting the findings of this study are available within the paper and its Supplementary Information files. Should any raw data files be needed in another format they are available from the corresponding author upon reasonable request.
