# Supplementary material for: Distribution of pharmaceuticals in marine surface sediment and macroalgae (ulvophyceae) around Mombasa peri-urban creeks and Gazi Bay, Kenya
Source: Environ Sci Pollut Res Int. 2025 Jan 24;32(7):4103–23. doi: 10.1007/s11356-024-35881-4 (PMC11835918; doi:10.1007/s11356-024-35881-4)
Supplement: Supplementary file 1 — Supplementary file1 (DOCX 970 KB) [file 11356_2024_35881_MOESM1_ESM.docx]

**Distribution of pharmaceuticals in marine surface sediments and macroalgae (ulvophyceae) around Mombasa peri-urban Creeks and Gazi Bay, Kenya**

Veronica Wayayi Ogolla Wanjeri ^1, 2^, Eric Okuku^2^, Jane Catherine Ngila^1^, Josephine Ouma^4^ and Patrick Gathura Ndungu^3^*

^1^ Department of Chemical Sciences, University of Johannesburg, Johannesburg, South Africa

^2^Kenya Marine and Fisheries Research Institute, P.O. Box 81651, Mombasa, Kenya

^3^Department of Chemistry, University of Pretoria, Hatfield, Pretoria, South Africa

^4^Jomo Kenyatta University of Agriculture and Technology, P.O. Box 62000, Nairobi, Kenya

| **Tudor (Dry season)**  **** | **Tudor (Wet season)**  **** |
| --- | --- |
| **Makupa creek (Dry season)**  **** | **Makupa creek (Wet season)**  **** |
| **Mtwapa creek (Dry season)**  **** | **Mtwapa creek (Wet season)**  **** |
| **Gazi bay (Dry season)**  **** | **Gazi bay (Wet season)**  **** |

**Figure S1: Relationships between measured physicochemical parameters and pharmaceuticals acetaminophen (ACN), acetylsalicylic (ASA), diclofenac (DCF), ibuprofen (IBF), trimethoprim (TMP), sulfamethoxazole (SMZ), tetracycline (TC), erythromycin (ERM), carbamazepine (CBZ), nevirapine (NVP), caffeine (CAF), cetirizine (CTN), lidocaine (LCN), bupivacaine (BCN) in surface sediment of Tudor creek, Mtwapa creek, Makupa creek, and Gazi bay [significant values 0.05, = (*), 0.01= (**) and 0.001= (***)].**

**Table S1: Risk quotients in the dry season for the pharmaceutical residues detected in surface sediment of Tudor, Makupa, Mtwapa creeks, and Gazi bay****. Potential ecological risks of sedimentary pharmaceuticals were classified into no risk (Log10 RQ < −2), low risk (−2 < Log10 RQ < −1), medium risk (−1 < Log10 RQ < 0), and high risk (Log10 RQ > 0) according to the Technical Guidance Document on Risk Assessment.**

| **Compounds** | **Trophic level** | **Organism/Species** |  | **Mean MEC (µg/kg)** | | | | **RQ (in Dry season)** | | | |
| --- | --- | --- | --- | --- | --- | --- | --- | --- | --- | --- | --- |
|  |  |  | **PNEC water (µg/ L)** | **Tudor** | **Makupa** | **Mtwapa** | **Gazi** | **Tudor** | **Makupa** | **Mtwapa** | **Gazi** |
| **Acetaminophen** | Fish | *Oryzias latipes* | 160 | 28.93 | 56.58 | 11.42 | 12.69 | 1.8 | 2.1 | 1.4 | 1.4 |
|  | Crustacea | *Artemia salina* | 100 |  |  |  |  | 2.0 | 2.3 | 1.6 | 1.6 |
|  | Algae | *Phaeodactylum tricornutum* | 265.8 |  |  |  |  | 1.6 | 1.9 | 1.2 | 1.2 |
| **Sulfamethoxazole** | Fish | *Oryzias latipes* | 750 | 0.27 | 1.24 | 0.19 | 0.10 | -1.3 | -0.7 | -1.5 | -1.7 |
|  | Crustacea | *Artemia salina* | 100 |  |  |  |  | -0.5 | 0.2 | -0.6 | -0.9 |
|  | Algae | *Skeletonema marinoi* | 5.35 |  |  |  |  | 0.8 | 1.5 | 0.7 | 0.4 |
| **Trimethoprim** | Fish | *Oryzias latipes* | 100 | 5.80 | 2.29 | 0.89 | 2.91 | 0.9 | 0.4 | 0.04 | 0.6 |
|  | Crustacea | *Artemia salina* | 100 |  |  |  |  | 0.9 | 0.4 | 0.04 | 0.6 |
|  | Algae | *Phaeodactylum tricornutum* | 5.1 |  |  |  |  | 2.1 | 1.7 | 1.3 | 1.8 |
| **Carbamazepine** | Fish | *Oryzias latipes* | 35.4 | 25.64 | 5.79 | 11.65 | 3.61 | 0.4 | -0.2 | 0.1 | -0.4 |
|  | Crustacea | *Artemia salina* | 100 |  |  |  |  | -1.0 | -1.7 | -1.4 | -1.9 |
|  | Algae | *Phaeodactylum tricornutum* | 62.5 |  |  |  |  | 0.2 | -0.5 | -0.2 | -0.7 |
| **Nevirapine** | Fish | Fish | 0.621 | 0.23 | 5.39 | 0.72 | 1.37 | 0.1 | 1.4 | 0.6 | 0.8 |
|  | Daphnia | Daphnia | 1.621 |  |  |  |  | -0.4 | 1.0 | 0.1 | 0.4 |
|  | Algae | Algae | 3.523 |  |  |  |  | -0.7 | 0.7 | -0.2 | 0.1 |

**Table S2: Risk quotients in the wet season for the pharmaceutical residue detected in surface sediment of Tudor, Makupa, Mtwapa creeks, and Gazi bay. Potential ecological risks of sedimentary pharmaceuticals were classified into no risk (Log10 RQ < −2), low risk (−2 < Log10 RQ < −1), medium risk (−1 < Log10 RQ < 0), and high risk (Log10 RQ > 0) according to the Technical Guidance Document on Risk Assessment.**

| **Compounds** | **Trophic level** | **Organism/Species** |  | **Mean MEC (µg/kg)** | | | | **RQ (in Wet season)** | | | |
| --- | --- | --- | --- | --- | --- | --- | --- | --- | --- | --- | --- |
|  |  |  | **PNEC water (µg/ L)** | **Tudor** | **Makupa** | **Mtwapa** | **Gazi** | **Tudor** | **Makupa** | **Mtwapa** | **Gazi** |
| **Acetaminophen** | Fish | *Oryzias latipes* | 160 | 14.09 | 12.25 | 17.30 | 10.48 | 1.5 | 1.4 | 1.6 | 1.4 |
|  | Crustacea | *Artemia salina* | 100 |  |  |  |  | 1.7 | 1.6 | 1.8 | 1.6 |
|  | Algae | *Phaeodactylum tricornutum* | 265.8 |  |  |  |  | 1.3 | 1.2 | 1.4 | 1.1 |
| **Sulfamethoxazole** | Fish | *Oryzias latipes* | 750 | 0.09 | 0.22 | 0.10 | 0.18 | -1.8 | -1.4 | -1.8 | -1.5 |
|  | Crustacea | *Artemia salina* | 100 |  |  |  |  | -0.9 | -0.6 | -0.9 | -0.6 |
|  | Algae | *Skeletonema marinoi* | 5.35 |  |  |  |  | 0.3 | 0.7 | 0.4 | 0.6 |
| **Trimethoprim** | Fish | *Oryzias latipes* | 100 | 1.33 | 1.50 | 0.16 | 3.36 | 0.2 | 0.3 | -0.7 | 0.6 |
|  | Crustacea | *Artemia salina* | 100 |  |  |  |  | 0.2 | 0.3 | -0.7 | 0.6 |
|  | Algae | *Phaeodactylum tricornutum* | 5.1 |  |  |  |  | 1.5 | 1.6 | 0.6 | 1.9 |
| **Carbamazepine** | Fish | *Oryzias latipes* | 35.4 | 8.73 | 3.56 | 7.28 | 17.88 | -0.1 | -0.4 | -0.1 | 0.3 |
|  | Crustacea | *Artemia salina* | 100 |  |  |  |  | -1.5 | -1.9 | -1.6 | -1.2 |
|  | Algae | *Phaeodactylum tricornutum* | 62.5 |  |  |  |  | -0.3 | -0.7 | -0.4 | 0.01 |
| **Nevirapine** | Fish | Fish | 0.62 | 0.62 | 5.98 | 0.43 | 0.53 | 0.5 | 1.5 | 0.3 | 0.4 |
|  | Daphnia | Daphnia | 1.62 |  |  |  |  | 0.1 | 1.1 | -0.1 | 0.0 |
|  | Algae | Algae | 3.52 |  |  |  |  | -0.3 | 0.7 | -0.4 | -0.3 |
